# Sasang constitution may act as a risk factor for prehypertension

**DOI:** 10.1186/s12906-015-0754-9

**Published:** 2015-07-14

**Authors:** Eunsu Jang, Younghwa Baek, Yunyoung Kim, KiHyun Park, Siwoo Lee

**Affiliations:** Department of Diagnosis, College of Korean Medicine, Daejeon University, 62 Daehak-ro, Dong-gu, Daejeon, 300-716 South Korea; Mibyeong Research Center, Korea Institute of Oriental Medicine, 1672 Yuseongdae-ro, Yuseong-gu, Daejeon, 305-811 South Korea; Department of Nursing, Sangmyung University, 31, Sangmyungdae-gil, Dongnam-Gu, Cheonan-si, Chungcheongnam-do 330-720 South Korea

**Keywords:** Sasang constitution, Prehypertension, Risk factor, Prevalence

## Abstract

**Background:**

Prehypertension (pre-HTN), similar to hypertension, has been associated with an elevated risk of cardiovascular disease. The aim of this study was to determine whether the Sasang constitution (SC) types could also be independent risk factors for pre-HTN.

**Methods:**

A total of 2,806 eligible subjects, older than 20 years old from 25 medical clinics, participated. Clinical data, including the blood pressure, age, height, weight, and data from blood tests, were collected. One-way ANOVA with Scheffé’s post-hoc analysis and the chi-square test were used, according to the SC and sex. Logistic regression was used to generate the odds ratios (ORs) and 95 % confidence interval (CI) for pre-HTN.

**Results:**

The pre-HTN prevalence rates of the Soeumin type, Soyangin type and Tae-eumin type were 59.4 %, 60.1 % and 74.9 %, respectively, in men (*p* < 0.001) and 41.7 %, 44.4 % and 58.3 % in women (*p* < 0.001). The Soyangin type was not more associated with increased ORs than the Soeumin type in any of the subjects with pre-HTN. Even after adjusting for sex, BMI, FBG, TC, TGs, HDL, and LDL, the Tae-eumin type in men was associated with increased ORs of pre-HTN compared with the Soeumin type (OR 1.57, 95 % CI 1.03–2.39), but the Tae-eumin type in women was not associated with pre-HTN.

**Conclusions:**

This study suggested that the Tae-eumin type combined with sex might be significantly and independently associated with pre-HTN, especially high pre-HTN.

## Background

Hypertension (HTN) is one of main risk factors for cardiovascular diseases, such as myocardial infarction, stroke, and heart failure, which increase both mortality and morbidity [1, 2]. Previous studies have revealed that blood pressure in the pre-hypertensive range might be associated with adverse cardiovascular outcomes [3]. High-normal blood pressure has been consistently associated with an elevated risk of cardiovascular disease [4, 5]. This association suggests that those with a prehypertension (pre-HTN) blood pressure are prone to progress to HTN [6]. Therefore, it has become important to manage health starting from the pre-HTN stage. The Seventh Report of the Joint National Commission (JNC 7) defined the range of HTN as systolic blood pressure (SBP) greater than 140 mmHg or diastolic blood pressure (DBP) greater than 90–99 mmHg [7]. It also defined the range for pre-HTN as 120 to 139 mmHg for SBP or 80 to 89 mmHg for DBP, defining a new risk category as a warning sign for developing HTN [7], with a low-end threshold for pre-HTN that is lower than the previous designation of high-normal blood pressure from the JNC 6 guidelines of 120 to 130 mmHg for SBP and from 80 to 85 mmHg for DBP [8].

The number of people with uncontrolled HTN and pre-HTN in the world has increased [9, 10] as the population has increased and aged [11]. The prevalence of uncontrolled HTN in Koreans is 22.9 % (26.9 % in men and 20.5 % in women), and the prevalence of pre-HTN was higher, at 31.6 % (41.9 % in men and 25.9 % in women), in 2001 [12]. Although the main causes of HTN and pre-HTN are unknown in most cases, genetic and environmental factors, such as sex, age, ethnicity, education, obesity, and several diseases, are suspected, in Western medicine, to be important causes of HTN and pre-HTN [3, 13].

Sasang constitutional medicine (SCM) is a type of Korean personalized traditional medicine that classifies people into the following four types: Taeyangin type (TY type), Soyangin type (SY type), Tae-eumin type (TE type), and Soeumin type (SE type) [14]. Each Sasang constitution (SC) type has a different susceptibility to physio-pathology and several diseases [15]. Several studies have suggested that the SC types could be risk factors for certain chronic diseases, including diabetes mellitus (DM), abdominal obesity, metabolic syndrome, functional dyspepsia, obstructive sleep apnea, and subclinical hypothyroidism [16–22]. The WONCA International Classification Committee defined chronic disease as long in duration, often with a long latency period and protracted clinical course; having a multi-factorial etiology; lacking a definite cure; and as gradually changing over time with an asynchronous evolution and heterogeneity in the population susceptibility [23]. The World Health Organization reported that obesity, diabetes mellitus, cardiovascular disease, HTN, stroke, and some cancer types that are related to dietary and lifestyle patterns are included in non-communicable diseases [24, 25].

From a preventive standpoint, the SC type is also considered to be crucial to the pre-disease and disease stages [14]. Therefore, it is also important to determine whether the SC type is a clinically important risk factor for the pre-disease stage. Though trends in Western medicine have recently focused on preventing disease, SCM in Korean medicine has emphasized preventing and predicting disease for a much longer period of time [14]. However, few studies have found associations between the pre-disease states and SC types, except for a study on abnormal glucose tolerance [17]. Recently, Lee’s study suggested that the SC types could be risk factors for HTN [26]. However, their study did not reveal SC to be a risk factor for pre-HTN. The aim of the present study was to present evidence for whether the SC types could be independent risk factors for pre-HTN.

## Methods

### Subjects

**Fig. 1:**
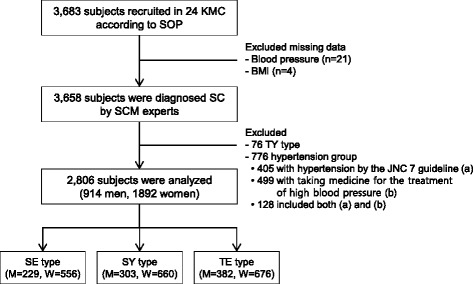
Flow chart of the subject enrollment. SOP: standard operating procedure, BMI: body mass index, SC: Sasang constitution, SCM: Sasang constitutional medicine, TY: Taeyangin, SE: Soeumin, SY: Soyangin, TE: Tae-eumin, M: men, and W: women

This study was conducted from November 2006 to July 2013 in South Korean medical hospitals. Participants older than 20 years old were recruited among the people who visited the hospital for medical examinations. Participants who could not understand or follow the researchers’ instructions due to severe physical and mental conditions, such as terminal cancer, were excluded. Pregnant women were also excluded. A total of 3,683 subjects (1312 men and 2371 women) enrolled in this study, but 25 were excluded because of missing data (21 for blood pressure data and 4 for body mass index data). Because the TY type is fairly rare in Korea, 76 TY types were excluded from this study. Additionally, because this study focused on pre-HTN, 776 patients with HTN (365 men and 411 women), according to the guidelines of the JNC 7 of ≥ 90 mmHg for DBP or ≥ 140 mmHg for SBP (405 subjects), or who were taking medicine to treat high blood pressure (409 subjects) were also excluded from this study; one hundred twenty-eight subjects were included in both of these categories. A total of 2,806 subjects (914 men and 1892 women) met the inclusion criteria. A detailed flowchart of this study design is shown in Fig. 1. The Korea Institute of Oriental Medicine Institutional Review Board approved of this study (KIOM IRB; number I-0910/02-001), and written informed consent was obtained from each subject. A flow chart of the subject enrollment is shown in Fig. 1.

### Data collection

Blood pressure was measured from the subjects’ left upper arms. To obtain stable blood pressure readings, the subjects were asked to rest before the measurements. The subjects’ height and weight were measured and rounded to the nearest hundredth, and the body mass index (BMI) was indirectly calculated from the height and weight. Other information, including the sex and age, and blood samples were collected to control for the confounding influences of important risk factors for pre-HTN. In particular, blood samples were collected after more than 12 h of fasting, and the fasting blood glucose (FBG), total cholesterol (TC), triglyceride (TGs), and HDL and LDL cholesterol were tested at one authorized institution (Seoul Medical Science Institute, SCL).

The instructors at each hospital met and were educated about the standardization of the data at least once per year, and KIOM continued to monitor the process of data collection several times per year. All of the instructors followed a standard operating procedure (SOP) that was developed for the “Korea Constitution Multicenter Study” [27].

### Diagnosis of the Sasang constitution

For a more accurate diagnosis of the Sasang constitution types, we confirmed three conditions according to a previous study [28]. First, the qualifications of the experts were strictly limited. Those experts, who had more than 5 years of clinical experience in SCM practice, participated in this study at South Korean national hospitals. They classified the subjects’ SC types based on their temperament, body shape, appearance, voice and physio-pathological symptoms. Generally, the inter- and intra-observer validities among the experts were more than 60 % [29]. Second, the medical charts of the participants were reviewed to determine whether they had taken herbal medicine for more than one month. An additional method of confirming the participants’ SC types was performed by assessing the participants’ health for SC-specific pharmaceuticals because unsuitable herbal prescriptions can cause adverse effects. Therefore, only those subjects who showed good improvement with SC-specific pharmaceuticals were candidate subjects. Last but not least, qualified SCM experts re-diagnosed the participants’ SC types.

### Diagnosis of pre-HTN

Pre-HTN was diagnosed according to the JNC 7 as 120–139 mmHg for SBP or 80–89 mmHg for DBP [7]. We classified the subjects into two groups and evaluated them according to European guidelines as follows: low pre-HTN (SBP 120–129 mmHg and/or DBP 80–84 mmHg) and high pre-HTN (SBP 130–139 mmHg and/or DBP 85–89 mmHg) [30].

### Statistical analyses

All of the data were analyzed separately according to sex. One-way ANOVA with Scheffé’s post-hoc analysis was conducted to compare the continuous variables. The chi-square test was used to compare the prevalence of pre-HTN according to the SC type. Multinorminal logistic regression was used to generate the odds ratios (ORs) and 95 % confidence intervals (CIs) for normal, low and high pre-HTN groups and binary logistic regression was used for normal and pre-HTN groups. To calculate the ORs, the risk of low pre-HTN (from 120 to129 mmHg for SBP or 80 to 84 mmHg for DBP), high pre-HTN (from 130 to139 mmHg for SBP or 85 to 89 mmHg for DBP) and pre-HTN (from 120 to 139 mmHg for SBP or 80 to 89 mmHg for DBP) according to the SC types were set and compared with the normal (below 120 mmHg for SBP and 80 mmHg for DBP) group. Covariant variables, such as age, BMI, fasting blood glucose, total cholesterol, triglycerides, and LDL and HDL cholesterol, were adjusted to evaluate whether the SC types could be risk factors for pre-HTN. The statistical level of significance was set at a *p* value < 0.05 using the SPSS software, version 21.0 (SPSS Inc., Chicago, IL, USA).

## Results

### General characteristics

The numbers of participants in the SE, SY and TE types were 229, 303 and 382 men, respectively, and 556, 660 and 676 women. The general characteristics, including age and BMI, were significantly different among the SC types. In detail, the SBP and DBP differed according to the SC types, and blood pressure was highest for the TE type. Other general characteristics, such as the BMI, FBG, TC, TGs, and LDL, but not height or HDL level, which are known potential risk factors for HTN, were also highest for the TE type. The details are described in Table 1.

**Table 1:** General characteristics of men and women according to Sasang constitution

Variables	Men				Women			
	SE type	SY type	TE type	*p* value	SE type	SY type	TE type	*p* value
N (%)	229 (25.1)	303 (33.2)	382 (41.8)		556 (29.4)	660 (34.9)	676 (35.7)	
Age	45.3 ± 14	50.4 ± 14.3	50.3 ± 14.8	<0.001 ^a, b^	45.4 ± 14.5	46.6 ± 13.5	50 ± 15.1	<0.001 ^b, c^
Height	169.9 ± 5.9	169.2 ± 5.9	170.2 ± 6.4	0.08	158.5 ± 5.7	157.3 ± 5.8	158 ± 5.9	0.001 ^a^
Weight	62.7 ± 7.9	66.5 ± 8.6	74.5 ± 10.7	<0.001 ^a, b, c^	52.4 ± 6.1	55.3 ± 6.8	62.3 ± 8.3	<0.001 ^a, b, c^
BMI	21.7 ± 2.3	23.2 ± 2.5	25.7 ± 2.9	<0.001 ^a, b, c^	20.9 ± 2.4	22.4 ± 2.7	25 ± 3.1	<0.001 ^a, b, c^
SBP	114.1 ± 11.2	116.3 ± 9.5	119.2 ± 8.8	<0.001 ^a, b, c^	111.7 ± 11.3	112.4 ± 11.1	115.3 ± 11.1	<0.001 ^b, c^
DBP	72.7 ± 9.2	74.1 ± 7.5	75.8 ± 7	<0.001 ^b, c^	71.4 ± 8.1	71.8 ± 8	73.8 ± 8	<0.001 ^b, c^
FBG	96 ± 27.1	102 ± 28.1	101.9 ± 27.4	0.021 ^a, b^	91.7 ± 14.7	94.9 ± 25.8	98.2 ± 26.4	<0.001 ^b, c^
TC	178.5 ± 33.7	184.8 ± 35	184.4 ± 34	0.069	180.2 ± 32.7	187.6 ± 36.2	188.1 ± 34.9	<0.001 ^a, b^
TG	116.8 ± 63.5	152.5 ± 104.2	160.1 ± 95.5	<0.001 ^a, b^	93.4 ± 53.4	105.1 ± 60.6	123.4 ± 73.7	<0.001 ^a, b, c^
HDL	46 ± 11.2	43.8 ± 11	40.7 ± 10	<0.001 ^b, c^	53.3 ± 12.7	50.8 ± 12.6	47.9 ± 11.7	<0.001 ^a, b, c^
LDL	103.4 ± 28.9	107.4 ± 30	108.1 ± 28.9	0.139	100.4 ± 28.7	108.4 ± 32.5	109.9 ± 30.5	<0.001 ^a, b^

Data shown are the means ± SDs or numbers

^a^Soeumin and Soyangin differ significantly; ^b^Soeumin and Tae-eumin differ significantly; and ^c^Soyangin and Tae-eumin differ significantly

*SE*: Soeumin, *SY*: Soyangin, *TE*: Tae-eumin, *BMI*: body mass index, *SBP*: systolic blood pressure, *DBP*: diastolic blood pressure, *FBG*: fasting blood glucose, *TC*: total cholesterol, *TG*: triglyceride, *HDL*: HDL cholesterol, and *LDL*: LDL cholesterol

### Prevalence of pre-HTN status by Sasang constitution

**Fig. 2:**
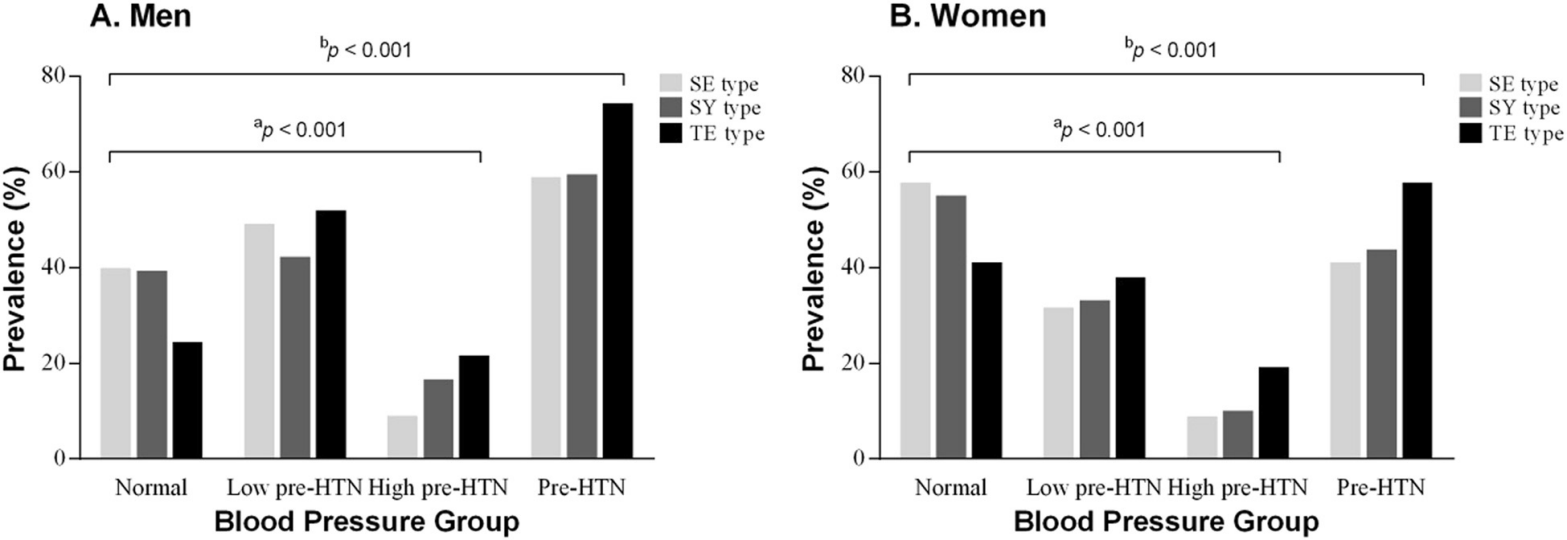
Prevalence of pre-HTN status by Sasang constitution. ^a^ *p* value, chi-square for normal, low and high pre-HTN among SC types in each men (**A**) and women (**B**); ^b^ *p* value, chi-square for normal and pre-HTN among SC types in each men (**A**) and women (**B**); SE, Soeumin; SY, Soyangin; TE, Tae-eumin; HTN, Hypertension

The prevalence rates of low pre-HTN among the SE, SY and TE types were 49.8 %, 42.9 % and 52.6 % in men, respectively, and 32.2 %, 33.8 % and 38.5 % in women. The prevalence rates of high pre-HTN among the SE, SY and TE types were 9.6 %, 17.2 % and 22.3 % in men (*p* < 0.001), respectively, and 9.5 % 10.6 % and 19.8 % in women (*p* < 0.001). In total, the pre-HTN prevalence rates of SE, SY and TE was 59.4 %, 60.1 % and 74.9 % in men (*p* < 0.001), respectively, and 41.7 %, 44.4 % and 58.3 % (*p* < 0.001) in women. There was a significant difference in the pre-HTN prevalence between the normal and pre-HTN groups (both the low and high pre-HTN groups) in both men and women. The details are shown in Fig. 2.

### Adjusted odds ratios for pre-HTN according to the SC types

Because the comparatively higher SBP and DBP might be influenced by age, BMI, and blood test results, it is necessary to demonstrate the pure risk value of the SC type alone. Table 2 presents the ORs for low pre-HTN, high pre-HTN and pre-HTN according to the SC type.

**Table 2:** Adjusted odds ratios and 95 % CIs for pre-HTN according to Sasang constitution

	Men			Women		
	SE	SY	TE	SE	SY	TE
Low pre-HTN						
Crude	Ref.	0.88 (0.61–1.27)	1.71 (1.18–2.46)*	Ref.	1.1 (0.86–1.41)	1.67 (1.3–2.14)**
Model 1	Ref.	0.86 (0.59–1.24)	1.67 (1.16–2.42)*	Ref.	1.06 (0.82–1.36)	1.51 (1.17–1.94)*
Model 2	Ref.	0.78 (0.53–1.14)	1.31 (0.85–2.01)	Ref.	0.88 (0.68–1.15)	0.93 (0.69–1.26)
Model 3	Ref.	0.78 (0.53–1.15)	1.36 (0.88–2.12)	Ref.	0.87 (0.66–1.13)	0.92 (0.68–1.25)
High pre-HTN						
Crude	Ref.	1.82 (1.03–3.2)*	3.74 (2.16–6.48)**	Ref.	1.17 (0.79–1.72)	2.9 (2.04–4.15)**
Model 1	Ref.	1.62 (0.92–2.88)	3.38 (1.94–5.88)**	Ref.	1.12 (0.75–1.67)	2.42 (1.67–3.5)**
Model 2	Ref.	1.43 (0.8–2.57)	2.43 (1.3–4.55)*	Ref.	0.94 (0.62–1.41)	1.53 (1–2.32)*
Model 3	Ref.	1.4 (0.78–2.54)	2.57 (1.36–4.84)*	Ref.	0.91 (0.6–1.38)	1.48 (0.97–2.28)
Pre-HTN						
Crude	Ref.	1.03 (0.72–1.46)	2.04 (1.43–2.89)**	Ref.	1.11 (0.89–1.4)	1.95 (1.55–2.45)**
Model 1	Ref.	0.98 (0.69–1.4)	1.95 (1.37–2.78)**	Ref.	1.07 (0.84–1.36)	1.7 (1.34–2.16)**
Model 2	Ref.	0.88 (0.61–1.27)	1.5 (0.99–2.26)	Ref.	0.89 (0.7–1.14)	1.07 (0.81–1.41)
Model 3	Ref.	0.88 (0.61–1.28)	1.57 (1.03–2.39)*	Ref.	0.88 (0.68–1.12)	1.05 (0.79–1.39)

*p < 0.05 **p < 0.001

Model 1 adjusted for age

Model 2 adjusted for age and BMI

Model 3 adjusted for age, BMI, FBG, total cholesterol, triglyceride, and LDL and HDL cholesterol

low pre-HTN: SBP, 120 to 129 mmHg and/or DBP, 80 to 84 mmHg, high pre-HTN: SBP, 130 to 139 mmHg and/or DBP, 85 to 89 mmHg

The ORs of TE type for low pre-HTN was significantly higher compared with the SE type in both crude (OR 1.71, 95 % CI 1.18–2.46 in men, OR 1.67, 95 % CI 1.3–2.14 in women) and model 1 (OR 1.67, 95 % CI 1.16–2.42 in men, OR 1.51, 95 % CI 1.17–1.94 in women). However, it showed no significance in model 2 and model 3 after adjusting for BMI variables. The ORs of TE type for high pre-HTN was significantly higher compared with the SE type in crude (OR 3.74, 95 % CI 2.16–6.48 in men, OR 2.9, 95 % CI 2.04–4.15 in women), model 1 (OR 3.38, 95 % CI 1.94–5.88 in men, OR 2.42, 95 % CI 1.67–3.5 in women) and model 2 (OR 2.43, 95 % CI 1.3–4.55 in men, OR 1.53, 95 % CI 1.–2.32 in women). However, the ORs of TE type for high pre-HTN was significantly higher compared with the SE type alone in men (OR 2.57, 95 % CI 1.36–4.84), after adjusting for all confounding variables in model 3.

The ORs of TE type for pre-HTN was significantly higher compared with the SE type in both the crude (OR 2.04, 95 % CI 1.43–2.89 in men, OR 1.95, 95 % CI 1.55–2.45 in women) and model 1 (OR 1.95, 95 % CI 1.37–2.78 in men, OR 1.7, 95 % CI 1.34–2.16 in women) but it was not significantly higher in model 2. The ORs of TE type for pre-HTN was significantly higher compared with the SE type alone in men (OR 1.57, 95 % CI 1.03–2.39) even after adjusting for age, BMI, FBG, TC, TGs, HDL and LDL, but it was not significant in women. The ORs of SY type for pre-HTN including low and high HTN was not significant compared with the SE type, except for high pre-HTN in crude in men.

## Discussion

This study focused on the SC type as a risk factor for pre-HTN, which is an important component of preventive medicine. This study suggested that the prevalence of pre-HTN in the TE type was highest in both men and women. However, as the comparatively higher SBP and DBP of the TE type might be influenced by the age, BMI, and several laboratory blood test results, the ORs for pre-HTN were calculated by logistic regression analysis, considering the influences of those potential variables. The TE type was associated with an increased OR of high pre-HTN in men compared with the SE type (OR 2.57, 95 % CI 1.36–4.84), but the TE type was not associated with an increased ORs in women after adjusting for the age, BMI, FBG, TC, TGs, and HDL and LDL cholesterol. This finding indicated that the TE types, combined with sex, might be significant and independent risk factors for pre-HTN as well as might work as predictive factors for pre-HTN, especially high pre-HTN.

In details, logistic regression analysis showed that the ORs of the TE type were significantly different from those of the SE type, in crude analysis, for both men and women. Because BMI is a well-known risk factor for pre-HTN [31], we tried to exclude both the BMI effect and other covariant variables through logistic regression analysis. The ORs of pre-HTN for the TE type was changed from 1.95 (*p* < .001, model 1) significantly to 1.5 (model 2) insignificantly and from 1.7 (*p* < .001, model 1) significantly to 1.07 (model 2) insignificantly in men and women compared with the SE type, respectively. However, the ORs of TE type in men for pre-HTN became significant again as 1.57 (*p* < .05) in model 3. From this view point not only BMI but also other covariant factors including SC seemed to affect to pre-HTN in men.

There was a different trend between men and women, wherein only the TE type in men was associated with an increased OR (1.57, 95 % CI 1.03–2.39) of pre-HTN compared with the SE type in model 3. These differences seemed to arise from sexual characteristics because the prevalence of pre-HTN is higher in men than in women [31–34]. Numerous studies have also shown that females have lower blood pressure levels throughout much of their lives compared with their age-matched counterparts. These studies have insisted that the relatively lower blood pressure levels in women are from the important role of sex hormones and different lifestyle, including smoking status [35–37].

The TE type in men had substantially increased ORs for high pre-HTN in particular, [38, 39]. The new category, so-called pre-HTN (120 to 139 mmHg systolic or 80 to 89 mmHg diastolic) was included in the JNC 7 guidelines because HTN is strongly related to CVD [7]. The recent 2013 European Society of Hypertension/European Society of Cardiology (ESH/ESC) guidelines for HTN proposed seven categories of BP levels, dividing pre-HTN into two categories, high pre-HTN (defined as a systolic BP of 130–139 mm Hg and/or diastolic BP of 85–89 mm Hg) and low pre-HTN (defined as a systolic BP of 120–129 mm Hg and/or diastolic BP of 80–84 mm Hg). High pre-HTN is associated with an increased risk of developing HTN as well as cardiovascular disease, including atrial fibrillation, but low pre-HTN has not been shown to be associated with an increased risk of cardiovascular disease. As a result, active management of lifestyle changes is necessary at the high pre-HTN stage. That’s why pre-HTN was divided into low and high pre-HTN. The TE type was significantly associated with an increased OR (2.43) in model 2 and OR (2.57) in model 3 for high pre-HTN, but it was not significantly associated with the OR (1.31) in model 2 and OR (1.36) in model 3 for low pre-HTN compared with the SE type, in men.

This result may be less conclusive because here, pre-HTN is simply the early stage of HTN, in contrast to another study [26], which reported that the TE type could be a risk factor for HTN. However, the present study, focusing on preventive view point at the early normal stage of elevated blood pressure, suggests that the TE type might be a risk factor for the high pre-HTN category in men, so requires more careful attention for high pre-HTN

This study had several strengths. First off, local bias and measurement error of data may less affect to the result, because the participants in this study were recruited nationwide from South Korean medical centers, and the acquisition of data in this study was clarified by following SOPs [27]. Secondly, this study followed preventive trends, which is the latest fashion in health promotion, by focusing on pre-disease HTN stages. This finding might provide second-hand evidence for SCM that corresponds to the original preventive intention.

This study also had some limitations. Because of its cross-sectional design, we could not determine a cause-and-effect relationship between the SC type and pre-HTN; as a result, the level of evidence was relatively low. Although we considered the influences of potential variables, we did not control for all of the risk factors that influence pre-HTN. Furthermore, the blood pressure of the subjects was only measured once, which might not be in agreement with the European guidelines, where blood pressure is classified based on two or more readings taken on two or more separate occasions, with at least one week between the measurements. Accordingly, people with pre-HTN might be affected by the white coat, or patients with masked HTN were included or excluded.

Further studies with longer-term follow-up designs are needed to confirm SC as an important risk factor for pre-HTN.

## Conclusion

The study findings suggested that SC, particularly the TE type in men, might be significantly and independently associated with pre-HTN especially high pre-HTN, revealing that SC could be an important health factor in pre-HTN.
